# Automatization and self-maintenance of the O-GlcNAcome catalog: a smart scientific database

**DOI:** 10.1093/database/baab039

**Published:** 2021-07-19

**Authors:** Florian Malard, Eugenia Wulff-Fuentes, Rex R Berendt, Guillaume Didier, Stephanie Olivier-Van Stichelen

**Affiliations:** Department of Biochemistry, Medical College of Wisconsin, 8701 Watertown Plank Rd, Milwaukee, WI, USA; Department of Biochemistry, Medical College of Wisconsin, 8701 Watertown Plank Rd, Milwaukee, WI, USA; Department of Biochemistry, Medical College of Wisconsin, 8701 Watertown Plank Rd, Milwaukee, WI, USA; Optionizr SAS, 9 Allée Claude Monet, Levallois-Perret 92300, France; Department of Biochemistry, Medical College of Wisconsin, 8701 Watertown Plank Rd, Milwaukee, WI, USA

## Abstract

Post-translational modifications (PTMs) are ubiquitous and essential for protein function and signaling, motivating the need for sustainable benefit and open models of web databases. Highly conserved *O*-GlcNAcylation is a case example of one of the most recently discovered PTMs, investigated by a growing community. Historically, details about *O*-GlcNAcylated proteins and sites were dispersed across literature and in non-*O*-GlcNAc-focused, rapidly outdated or now defunct web databases. In a first effort to fill the gap, we recently published a human *O*-GlcNAcome catalog with a basic web interface. Based on the enthusiasm generated by this first resource, we extended our *O*-GlcNAcome catalog to include data from 42 distinct organisms and released the *O*-GlcNAc Database v1.2. In this version, more than 14 500 *O*-GlcNAcylated proteins and 11 000 *O*-GlcNAcylation sites are referenced from the curation of 2200 publications. In this article, we also present the extensive features of the *O*-GlcNAc Database, including the user-friendly interface, back-end and client–server interactions. We particularly emphasized our workflow, involving a mostly automatized and self-maintained database, including machine learning approaches for text mining. We hope that this software model will be useful beyond the *O*-GlcNAc community, to set up new smart, scientific online databases, in a short period of time. Indeed, this database system can be administrated with little to no programming skills and is meant to be an example of a useful, sustainable and cost-efficient resource, which exclusively relies on free open-source software elements (www.oglcnac.mcw.edu).

## Introduction

**Figure 1.**
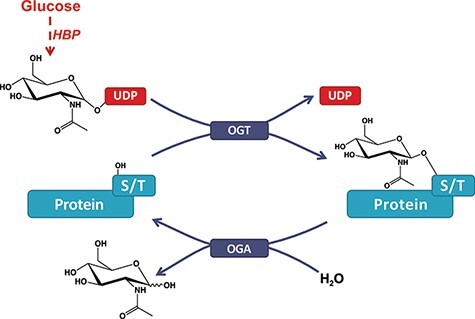
*O*-GlcNAcylation of proteins. A single β-*N*-acetyl-glucosamine residue is added by the *O*-GlcNAc transferase (OGT) and removed by the *O*-GlcNAcase (OGA). The hexosamine biosynthesis pathway drives the production of the *O*-GlcNAc nucleotide donor (e.g., UDP-GlcNAc) from glucose (Glc). Serine or threonine (S/T) is targeted for modification on intracellular proteins.

Protein post-translational modifications (PTMs) play an essential role in the biosynthesis of functional proteins and their signaling pathways. Consequently, defects in PTMs are associated with numerous pathological conditions and are excellent candidates for diagnosis (1) and therapy development (2). Along with well-established PTMs (e.g., phosphorylation), interest is growing toward more recently discovered PTMs, such as *O*-GlcNAcylation. This highly ubiquitous PTM is the addition of β-*N*-acetylglucosamine to the hydroxyl group of serine or threonine residues (3, 4) (Figure 1). While *O*-GlcNAcylation correlates with pathologies like Alzheimer’s disease, diabetes and cancers (5), the lack of an up-to-date bioinformatics resource slowed down its exposure to the greater scientific community. Indeed, the historical and very useful web database dbOGAP (6), first released in 2011, has now been defunct for years. More generally, PTM’s diverse and ubiquitous nature combined with the generalization of high-throughput methods has been a challenge for the development of sustainable databases, for which *O*-GlcNAcylation is the perfect example. As such, novel database models directed toward sustainability with minimum funding, human skills and time are key to maintain a reliable resource with the extended lifetime for the benefit of the community.

In a first effort to fill the gap, we recently published an initial catalog of the human *O*-GlcNAcome containing over 5000 proteins and 7000 sites (7). We made this catalog available on various platforms including the glycobiology platform GlyGen (8) and FigShare (9). Attached to this initial publication (7), we also included a basic interface. The *O*-GlcNAc Database Beta, released online at oglcnac.mcw.edu on 20 November 2021. By publishing our human *O*-GlcNAcome catalog, we rigorously confirmed that *O*-GlcNAcylation affected all classes of intracellular, mitochondrial and nuclear proteins, regulating their stability, activity, localization and other PTMs, such as phosphorylation (10). In general, *O*-GlcNAcylation is recognized as a highly conserved modification found in almost all living organisms, cells and tissues. Consequently, in light of the enthusiasm generated by the human catalog and web interface, we extended the content to cover all organisms and associated *O*-GlcNAcylated proteins. In parallel, important efforts have been assigned to the development of the *O*-GlcNAc Database to offer a sustainable resource with high reliability, minimal cost and extended lifetime. The current *O*-GlcNAc Database v1.2 long-term release is available at oglcnac.mcw.edu.

Herein, we outline the content provided by the *O*-GlcNAc Database, now including data for all available organisms besides human. Specifically, we emphasize the smart features of such database, e.g., smart organization, automatization and self-maintenance, while using the *O*-GlcNAc Database as a case study. We offer extensive software modeling diagrams of the system using the ISO standard Unified Modeling Language (UML) (11) and its derivative UML-based Web Engineering (UWE) (12) language. Notably, we detail our strategy for semi-automated literature curation using Machine Learning (ML) and Natural Language Processing (NLP) protocols to build a logistic binary classifier relying on neural networks (NNs). Finally, we release stand-alone tools derived from the *O*-GlcNAc Database source code, available through the python package utilsovs available at github.com/Synthaze/utilsovs/. (v0.9.1b).

In addition to serving the *O*-GlcNAc field, we hope that this work will enable other researchers to develop analogous database systems, focused on sustainability, by minimizing the need for funding, human skills and time. By providing detailed principles and methodological schemes for such systems, we draw a path for those who code for science and wish to offer an accessible resource to the scientific community.

## Methods

### Web server environment

The GNU/Linux (13, 14) distribution Debian 10 (Buster) was used to develop the *O*-GlcNAc Database, which relies exclusively on free and open-source software elements. For production, the *O*-GlcNAc Database server runs on the GNU/Linux (13, 14) distribution Ubuntu 18.04.5 LTS (kernel 4.15.0-122-generic x86_64) with the Ubuntu Server environment (ubuntu-server 1.417.5 x86_64). The *O*-GlcNAc Database was built with the Django web framework (3.7.1) (15), and MongoDB (mongodb-org 4.4.4) (16) was used for the back-end database. A UML (11) sequence diagram of the *O*-GlcNAc Database web request processing scheme is presented in Figure S1. Nginx (1.14.0) (17) was used as both reverse proxy and content delivery network. Upon user HTTPS request, Nginx (17) acts as a reverse proxy interface for the front-end server. Nginx Transport Layer Security (TLS 1.2) and Server Name Indication protocols enabled compliance with the HTTPS-only standard (18). The HTTP request is then forwarded to the Python HTTP server Gunicorn (19), which uses the WSGI (Web Server Gateway Interface) interface (20) to communicate with the Django application (15) and to serve users with dynamically generated content. Dynamic content upon Gunicorn request is generated by interrogating a back-end MongoDB database (16) via the PyMongo library (21), directly retrieving pre-calculated HTML code to optimize the server response time. Inversely, the generated dynamic content is passed through the Django application to be sent to the Gunicorn process, ultimately communicating the data to Nginx and the end user. Finally, the associated static content is served by Nginx over SSL to the end user upon GET request. The *O*-GlcNAc Database is available at both oglcnac.mcw.edu and oglcnac.com.

### Programming and libraries

The front-end interface was developed using HTML5 (22) and CSS3 (23) stylesheets with the framework Bootstrap (4.1.3) (24). On web pages, dynamic behaviors were implemented using the Javascript library JQuery (3.3.1) (25) and Django libraries for template tags and filters (built- in and custom). We used the World Wide Web Consortium (W3C) Markup Validation Service (26) to validate the web pages’ source code against HTML5 specifications. The back-end part of the *O*-GlcNAc Database was developed using Python (3.7.1) (27) and the high-level Python web framework Django (15). Our back-end architecture includes the web application back-end, directly supporting the front-end interface and a stand-alone library enabling automatization and self-maintenance of the *O*-GlcNAc Database. In addition to the Python standard library and Django package, the PyMongo (3.11.3) (21) library interacts with the MongoDB back-end database. All python libraries utilized by the *O*-GlcNAc Database system are reported in Table S1.

### Neural network

With the goal of automatically sorting the *O*-GlcNAc literature, each PDF file was extensively processed to be sorted based on the presence of *O*-GlcNAcylated proteins and site identifications using NNs, designed as an ensemble of logistic binary classifiers (28) (Figures S2–S4).

#### Text processing

We extracted text from publication PDF files using the command-line tool pdftotext from the .deb package poppler-utils (Figure S2, left panel). First, all expressions in text contained between brackets or parentheses as well as all non-space, non-period and non-alphanumerical characters were removed using regular expressions (**29**) in Python. The same processing was applied to a list of stop words compiled from general web resources and composed of names, journal names, chemicals, numerical and units, cities, countries and custom entries. Since PDF is a layout-based format which specifies the fonts and positions of the individual characters rather than the semantic units of the text (**30**), we used this list of stop words to detect and remove unwanted semantic units upon conversion (e.g., author names and affiliations, footnotes and tables). Then, we collected semantic units associated with results and discussion using a custom dictionary of regular expressions associated with sections usually found in publications (e.g., Introduction, Material and Methods and Results). To detect relevant vocabulary, we tagged words by category using the following lists: biology, glycobiology, cells and methods. With regular expressions, we tagged strings associated with the following categories: conclusion, description, peptides, nucleic acids, pronominial, ser/thr, amino acids, phosphorylation and *O*-GlcNAc. Protein and organism names were detected by matching dictionaries built from UniProtKB ftp repository against publication text files. We then removed all non-space, non-tag and non-period characters from text to finally retain period-separated strings containing more than one tag (Listing S1). All lists of words and regular expressions utilized for text labeling are provided in the supplementary data (Files S1–S3) with examples (Table S2).

#### Data sets and input preparation

Research articles (*n* = 1340) were each labeled as positive or negative depending on whether or not experimental demonstration of protein *O*-GlcNAcylation was found in a given publication. With the goal of using an NN designed as a logistic binary classifier, positive or negative labels were translated into binary labels such as (**1**,0) and (0,**1**), respectively, meaning that positive publications have a probability of 1 to contain experimental evidences of protein *O*-GlcNAcylation and a probability of 0 to not contain experimental evidences of protein *O*-GlcNAcylation. Training (*n* = 670), testing (*n* = 335) and validation (*n* = 335) sets were obtained by shuffling and slicing the list of PMID (PubMed IDentifier) identifiers (*n* = 1340) containing an equal number (*n* = 670) of positive and negative samples. To this extent, we used the random.shuffle() method without seed optimization to avoid bias. Sets were accepted if we found less than a 1% difference in the population of positive and negative labels in order to approximate a binomial distribution of labels within each set (Table S3). Text files corresponding to PMIDs within the whole training set (*n* = 670) were then concatenated, and period-separated strings, filtered by the number of occurrences to extract chains of tags or expressions (*n* = 1252), were represented at least four times in the whole training set to minimize artifacts during the logistic regression, particularly with the testing and validation sets. This list of expressions was matched against each publication text file (*n* = 1340), yielding a descriptor array for each publication composed of binary values (*n* = 1252) depending on the presence (**1**) or absence (0) of a given expression in a given file. The resulting arrays served as inputs for logistic binary classifiers (Figure S2, right panel). Training inputs were prepared to further proceed with bootstrap aggregation, often abbreviated as bagging, which involves having each model in the ensemble vote with equal weight (**31**). To this extent, training subsests (*n* = 5) were prepared by iteratively shuffling and slicing the list of PMIDs corresponding to the whole training set.

#### Architecture and training

A feed-forward (32) NN with backward propagation of errors (33) and dropout regularization (34) was developed from scratch to design a logistic binary classifier. The NN was composed of 1252 input units, one hidden layer with 96 rectifier linear units (35) and 2 sigmoid units in the output layer (Figure S3). Computations were performed using a standard laptop (i7- 7700HQ at 2.8 GHz ×8, 24GB SDDR4 RAM at 2400 MHz) running on the GNU/Linux distribution Debian 10 (Buster) (36) and using the numpy (1.20.1) (37) python library for its linear algebra and native multi-threading capabilities. For the training procedure, hyperparameters were first optimized using a grid-search procedure and were further adjusted manually. For the actual training procedure, we performed learning rate cycling (38) with an initial learning rate (ilr) of 0.25, an exponential decay rate (*k*) of 0.05 and a cycling constant (*c*) of 100 epochs. Starting cycle 2, the ilr was reduced using a descent coefficient (*d*) of 0.95 (Listing S2). The dropout regularization parameter was set to 0.7.

We performed training by fitting each training subset (*n* = 5) for 10 cycles (1000 epochs) following an early stopping procedure (39). The list of parameters then predicted the label series associated with each sample within the whole training, testing and validation sets (Figure S4). Label series were averaged for each sample, and uncertainties were calculated using a binomial probability formalism (Listing S3). For each sample, the aggregated prediction was considered as correct when rounded to the closest integer that corresponded to the true label associated with the sample. On the contrary, the aggregated prediction was considered as ambiguous or wrong if the absolute difference between the limits of the confidence interval was greater or lower than 0.5, respectively. Although all predictions are manually checked with inspection of extracted text and keywords, particular attention is dedicated to ambiguous predictions, which may more frequently require the inspection of the PDF file.

### Python package utilsovs

The utilsovs package (v0.9.1b) brings together tools derived from the *O*-GlcNAc Database source code and requires Python (≥3.7). Briefly, the utilsovs package contains a series of simple tools for programmatic access to major databases (e.g., UniProtKB, PubMed and SemanticScholar), for protein sequence digestion, for representation of alignment consensus as sequence logo (40) and for quality control of proteomic data sets along with other tools (Listing S4). The full documentation and the package can be found at github.com/Synthaze/utilsovs/ and pypi.org/project/utilsovs-pkg.

## Results

### Navigation and main features

**Figure 2.**
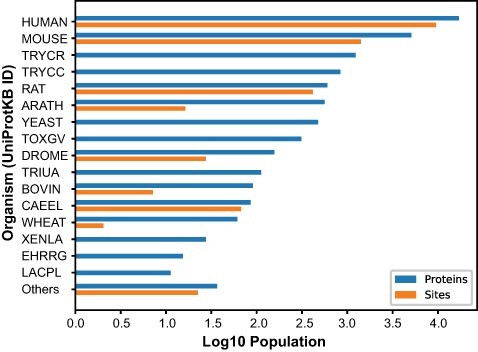
Number of *O*-GlcNAcylated proteins and sites. *O*-GlcNAcylated proteins (blue) and *O*-GlcNAc sites (orange) for each organism cataloged in the *O*-GlcNAc Database. The *Others* category summarized proteins and sites from 26 organisms with <10 protein entries.

The *O*-GlcNAc Database (oglcnac.mcw.edu/statistics) is a web resource based on literature curation that informs on the *O*-GlcNAc status of proteins across phyla using a simple and efficient navigation scheme (Figure S5). In the current release of the *O*-GlcNAc Database (v1.2), we documented the *O*-GlcNAcome of 42 distinct organisms. As its core feature, the *O*-GlcNAc Database (https://www.oglcnac.mcw.edu/search/) page provides a custom search engine matching the alphanumerical part of one request to relevant entry field counterparts in a case-insensitive manner, thus greatly improving tolerance toward mistyping on queries. Currently, 14 474 *O*-GlcNAcylated up-to-date canonical protein sequences and 11 182 *O*-GlcNAc sites can be accessed upon search (Figure 2, Table S5). These data are supported by the curation of 2169 *O*-GlcNAc publications, identified from PubMed’s default search with ‘*O*-GlcNAc’ as a query. These statistics can be accessed by navigating through the *O*-GlcNAcylation menu, which provides links toward the (https://www.oglcnac.mcw.edu/overview/) (general information), (https://www.oglcnac.mcw.edu/statistics/) (protein and literature entries, authors) and (https://www.oglcnac.mcw.edu/consensus/) (sequence logos) pages. In addition, metadata for the *O*-GlcNAc literature items we considered are available in the (https://www.oglcnac.mcw.edu/references/) page with search options (PMIDs and protein), filters (author and organism), sorting option (year, first/last author and organism) and selection of bibliography type (protein identification, research articles and reviews). The various data sets are available through the (https://www.oglcnac.mcw.edu/explore/) page, which displays the *O*-GlcNAcome with organism-specific filtering and sorting options (*O*-GlcNAc score, entry name, number of sites and organism name). For large experimental data sets (e.g., mass spectrometry), an advanced search mode is also provided, allowing the scientist to match a given data set against the *O*-GlcNAc Database with specific results, a visualization interface and custom reports generated in several formats. For offline comparison and analysis, users can download all or part of our data sets (protein and literature entries) via the (https://www.oglcnac.mcw.edu/download/) page. We encourage users to get involved in the *O*-GlcNAc Database weekly updates by submitting putatively missing *O*-GlcNAcylated proteins via the (https://www.oglcnac.mcw.edu/submit/) page or by addressing more general comments via the (https://www.oglcnac.mcw.edu/about/) page. Citation and version information about the *O*-GlcNAc Database can be found at the (https://www.oglcnac.mcw.edu/cite/) page. Finally, a REST API is available for programmatic access to the *O*-GlcNAc Database content for developers at (https://www.oglcnac.mcw.edu/api/v1/docs). To best suit user needs, options for data download are numerous across the *O*-GlcNAc Database and offer several formats for protein-related (CSV, XLSX, PDF and JSON) and literature-related (BIB, CSV, XLSX and JSON) data.

### Protein search and content

**Figure 3.**
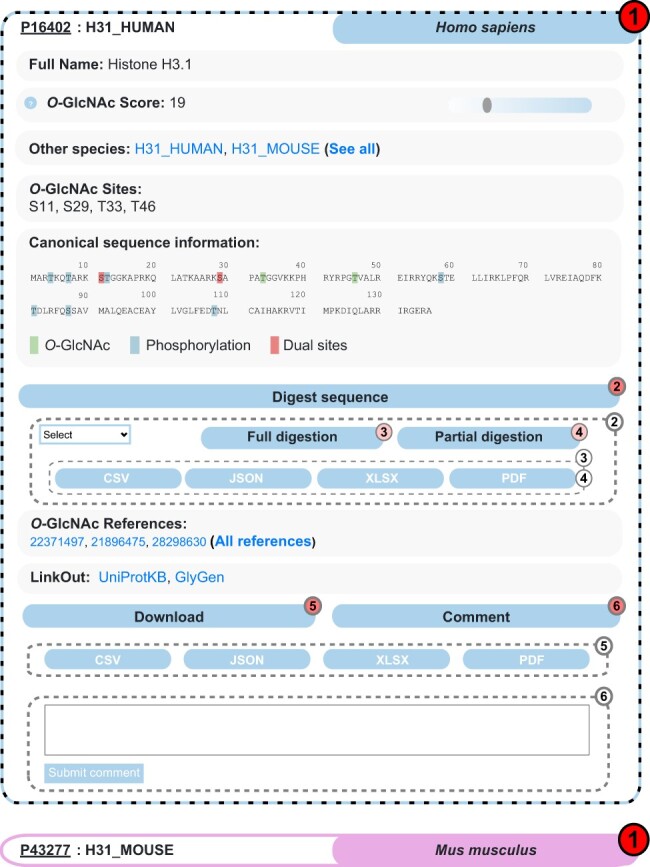
Example of search results for the protein Histone H3.1 in the *O*-GlcNAc Database. Protein entries are shown as collapsible elements (1), and child elements can be accessed on click (dashed frame). Nested collapsible provides digest tools (2) in full (3) and partial (4) modes, download (5) and comment options (6).

Interactive search for *O*-GlcNAcylated proteins is the core feature offered by the *O*-GlcNAc Database via the (https://www.oglcnac.mcw.edu/search/) page. Users can search for a single protein, a list of UniProtKB identifiers or even by matching a custom data set against the *O*-GlcNAc Database content. Upon search, the results are returned as a compact list of clickable, collapsible elements, each displaying essential information for a given protein (UniProtKB ID, entry name and organism) and following a color code specific to the parent organism (Figure 3). Upon click, uncollapsed elements show the essentials provided by the *O*-GlcNAc Database, including full protein name, *O*-GlcNAcylated orthologous proteins, *O*-GlcNAc score, reported *O*-GlcNAc sites and associated *O*-GlcNAc references. Because most *O*-GlcNAc scientists are interested in the interplay with phosphorylation, the protein sequence shows distinct highlights on *O*-GlcNAc sites, phosphorylation sites and dual sites. Users can easily jump to other major resources (UniProtKB and GlyGen) using the entry-specific links we provide. In addition to these essentials, one can navigate through the nested Digest sequence collapsible element, which enables full or partial protein digestion upon selection of protease and format for report generation. Entry-specific information can be downloaded upon click on the nested Download element and format selection. Finally, as user feedback is a priority for us, the nested Comment collapsible element allows anyone to discuss the current entry, which will be sent to us for review.

### Literature evaluation using ML

We used ML and developed a simple NN designed as a logistic binary classifier, aiming to predict whether *O*-GlcNAcylated proteins were experimentally validated in the chosen article (positive or negative). Preliminary to NN training, 1340 research articles (referred to as samples) were extensively processed from PDF format to text expression patterns. From this, binary inputs were generated for training (see the ‘Methods’ section). Briefly, heterogeneity from natural language and numerical data was reduced by translating text publications in lists of generic expressions patterns, thus diminishing the variance among positive and negative data sets (see, Listing S1). Then, 1252 patterns frequently detected in samples were isolated, against each of the 1340 samples, to prepare inputs as binary values for regression, depending on the presence (1) or absence (0) of a given pattern in a given sample (Figure S2, Table S2).

**Figure 4.**
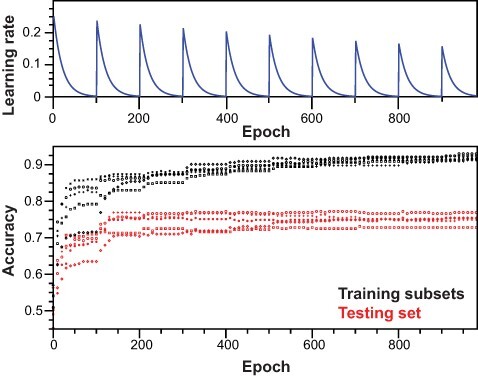
Training of the neural network. Top panel: scheme for learning rate cycling during training of independent models. Bottom panel: for each independent model, accuracy was monitored along training epochs for training subsets and testing set.

In order to improve model stability and accuracy impaired by the high variance inherent to natural language and small data sets, we proceeded with bootstrap aggregation, or bagging, of single learners (Figures S3 and S4). For each independent model, training subsets were generated by randomly selecting half of the training set. In the training procedure, model regression was exclusively driven by training data. Cyclical learning rate improved model accuracy (Figure 4, Listing S2), and we employed dropout regularization and early stopping to prevent overfitting. For early stopping, we saved weights and bias parameters that corresponded to the highest accuracy calculated on the testing set during the training procedure. Before bagging, all independent models could explain the positive and negative ensembles in the training subsets with accuracy >90% and no evidence of severe overfitting (Figure 4, Table 1). As expected, accuracy on the testing set for each independent model was lower, ranging from 73.1% to 77.5%. However, all models could be cross-validated by calculating the accuracy on the validation set, yielding values from 70.8% to 76.1%, consistent with the testing set. Upon bagging and prediction of positive and negative labels on the whole training set, an accuracy of 84.3% was obtained. Model accuracy was greatly improved for both testing and validation sets, with accuracies of 78.4% and 77.01%, respectively. Over the full data set (*n* = 1340), precision and recall were 81.87% and 77.81%, respectively. Finally, the accuracy reached 94.9% on the full training set when including samples for which the averaged decision was not correct but flagged as ambiguous. Following the same rule, the accuracy reached 88.6% and 87.2% for the testing and validation sets, respectively (Table 1), allowing to recover a greater fraction of relevant samples but with lower precision. Overall, we conclude that our aggregated model predicts the training data well, but most importantly, the testing and validation data (Table 1). We do not anticipate further improvements of the model, as we consider both intrinsic limitations (number of samples and natural language) and the requirement for human examination and validation of results in the context of the *O*-GlcNAc Database, in order to provide the best reliability level for the scientific community.

**Table 1.** Evaluation of model performance and cross-validation

Model	Iter.	Train. (+ Amb.)	Test (+ Amb.)	Val. (+ Amb.)	Epoch
1	1000	0.898	0.775	0.761	518
2	1000	0.909	0.731	0.743	710
3	1000	0.921	0.757	0.708	905
4	1000	0.863	0.775	0.755	211
5	1000	0.912	0.757	0.74	803
Agg. Pred.	NA	0.843 (0.949)*	0.784 (0.886)	0.7701 (0.872)	NA

The maximum number of iterations, the model accuracy measured on the actual training subset and the testing and validation set are shown for each model (1–5). Epoch corresponds to the iteration at which weight and bias matrix were saved. Upon bootstrap aggregation (Agg. Pred.), accuracy is shown for the entire training sets (*), as well as the testing and validation sets.

### Computer-aided literature curation

**Figure 5.**
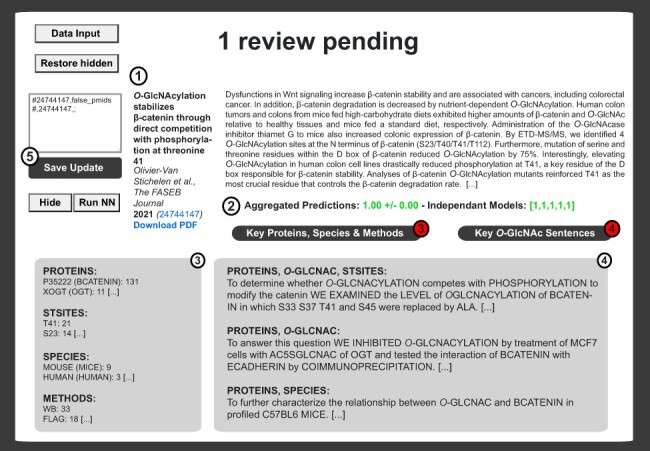
Example of literature report upon logistic binary classifier and automatization routines. The private interface contains the literature item metadata (Authors, Title, Year, Journal, Volume, Issue, Abstract, PMID and PubMed Link) (1) as well as the prediction score from the neural network (2). Neural network decisions are presented for each model ((2) right brackets). Decisions are then average and binomial confidence interval is calculated ((2) left). In (3), extracted proteins, sites, species and methods are shown next to the number of iteration for each item. This information is complemented by sentences associated with combinations of tags relevant to *O*-GlcNAcylated proteins (4). An update window is available for rapid update of the master update file upon inspection of each publication (5).

In addition to enhancing the user experience, the administrator experience was also improved. The main goal is to minimize maintenance time, while offering up-to-date and reliable content to the public. Therefore, PubMed is interrogated for new publications with the query ‘*O*-GlcNAc’ in default search on a weekly basis and requests the appropriate persons for full-text PDFs. Files are automatically processed to generate inputs from which the likeliness that each publication identifies a protein’s *O*-GlcNAcylation is computed using the aggregated logistic binary classifier model, described in the ‘Methods’ section and Literature evaluation using ML subsection. Intermediate files are then generated upon PDF processing to collect and count critical keywords, such as protein and species names or methods, as well as presumably informative sentences, including combinations of tags such as ‘PROTEIN, OGLCNAC, STSITES’. In the private section of the *O*-GlcNAc Database, this information is compiled with publication metadata in an admin-friendly interface for literature review (Figure 5). Upon PDF upload, the classifier can be immediately triggered on one click. Uploaded PDFs can be downloaded by lab members for a more detailed inspection. Pending reviews can be hidden and restored on demand. Finally, updates to the database can be implemented using dedicated text area, which takes simple CSV-formatted instructions, further considered by the automated update routine. Overall, this admin-friendly system minimized the time commitment (<2 min/article) associated with the maintenance of the database content.

### Self-maintenance and updates

**Figure 6.**
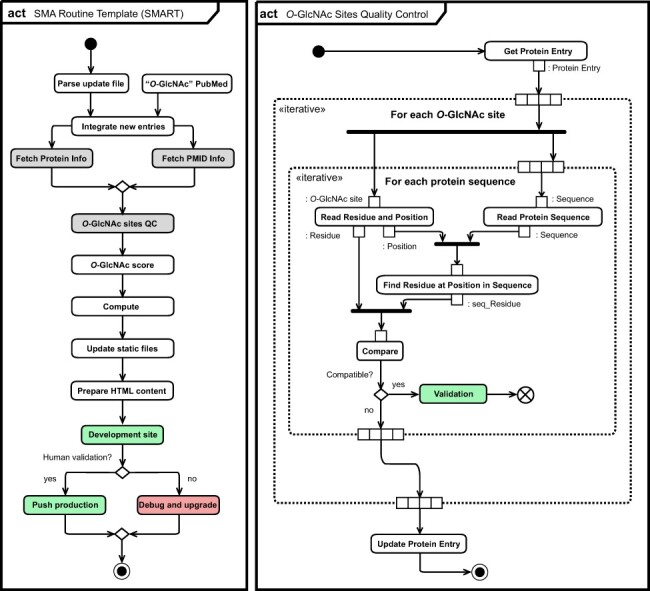
Unified Modeling Language (UML) (11) activity diagram (act) of the *O*-GlcNAc automatization and self-maintenance library. Initial state (black circle), actions (rounded rectangle), list objects (rectangle), fork and join (bold bars), decision and merge (diamond), break (crossed circle) as well as final state (black circle) are highlighted per UML conventions. Normal (green) and error (red) completion actions are also highlighted, together with actions for which specific activity diagrams for *O*-GlcNAc sites quality control (right panel) and for collection of information related to protein and literature are given (Figure S6).

Update of the *O*-GlcNAc Database runs automatically on a weekly basis (Figure 6). The routine starts by parsing the CSV-formatted instructions file introduced in the Computer-aided literature curation subsection (see, Listing S4). Upon update of MongoDB collections, the pipeline retrieves protein information from UniProtKB and PMID information from PubMed/MedLine and Semantic Scholar (41) (Figure S6). It also interrogates proteomeXchange (42) to link MS data sets associated with PMIDs. As previously introduced (43), quality control of *O*-GlcNAc site residue and position is performed against relevant protein sequences fetched from UniProtKB. The pipeline then computes all variable content found in the *O*-GlcNAc Database, including the *O*-GlcNAc score associated with each protein entry (43), *O*-GlcNAc Database statistics, *O*-GlcNAc site consensus, downloadable content for protein and literature (entries and data sets) and image files. Finally, the pipeline greatly optimizes the server response time by pre-calculating HTML code for essentially all content delivered to users, which also preserves clarity in the source code of the *O*-GlcNAc Database web interface. These automatic weekly updates are available locally on our development server, and lab members can trigger the export of the MongoDB back-end database to the production server upon inspection of changes and validation.

## Discussion

Back in 2011, the authors of the now defunct dbOGAP reported a total of 800 *O*-GlcNAcylated proteins, of which 500 were human proteins (44). A decade later, we document 14 474 *O*-GlcNAcylated proteins, which represents a growth of ∼1800%. Specifically, for humans, we reported 7 057 *O*-GlcNAcylated proteins, which represents an increase of ∼1450%. More importantly, this represents approximately one-third of all canonical proteins in the human proteome (45). As a comparison, the number of human phosphorylated proteins is around 10 456 (46), which represents about half of the human proteome (45). Finally, ∼50% of human *O*-GlcNAcylated proteins were also found to be phosphorylated, emphasizing the interplay between the two modifications. Although we are approaching the growth ceiling for the identification of new *O*-GlcNAcylated proteins in humans, this is far from true for other species. We also reported 7428 *O*-GlcNAc sites in human proteins, which is only ∼9% of the 86 181 phosphorylation sites reported in human proteins using comparable criteria (46). More than ever, these numbers highlight an obvious gap for the *O*-GlcNAc field.

By first publishing the human *O*-GlcNAcome catalog (43) and by extending it to 42 distinct species, we aim to fill the gap left by the defunct dbOGAP. To ensure the long-term success of this resource, we made the schemes we developed explicit in this work, by making the *O*-GlcNAc Database a sustainable resource, relying on extreme self-maintenance and automatization processes and still preserving the highest level of content reliability. Indeed, the *O*-GlcNAc Database merges both the computational and human worlds: calculation and workload for the first and capacity of judgment for the second. As such, every bit of content provided by the *O*-GlcNAc Database is and will always be validated by human interventions absolutely required at critical steps.

In addition to minimizing the need for human time and programming skills, the *O*-GlcNAc Database was made sustainable by lowering the need for funding over time. Accordingly, we had no funding to declare in the corresponding section of this manuscript. Although widely adopted for decades by informed individuals, we wish to emphasize that the near zero cost for this project is entirely made possible, thanks to the open-source community that develops and maintains world-leading software, such as the GNU/Linux family of operating systems, the Python programming language or the Django web framework. In addition to being free of charge, open-source software provides the following: the right to access the software’s source code, the right to make improvements to the program and the right to copy and to redistribute the original or modified program (47). Therefore, mature open-source technology is generally recognized as being a safer and more reliable alternative compared to proprietary counterparts, its organizational form massively encouraging critical peer review and the sharing of ideas (48, 49).

Finally, to make this system useful beyond the *O*-GlcNAc field, we developed a python package utilsovs containing general tools derived from the *O*-GlcNAc Database source code and encourage feedback to improve this resource. To conclude, we wish the *O*-GlcNAc database to be an example of reliable and sustainable scientific catalogue that inspire the development of many smart scientific database to come.

## Supplementary Material

baab039_Supp

## Data Availability

The *O*-GlcNAc Database is available online at oglcnac.mcw.edu and via our REST API for programatic access (https://www.oglcnac.mcw.edu/api/v1/docs/). Our data sets can be downloaded in several formats (CSV, JSON, XLSX, PDF and BIB). We also release the python package (https://github.com/Synthaze/utilsovs/) (v0.9.1b), which brings together utils derived from the *O*-GlcNAc Database source code.
