# Supplementary material for: Automatization and self-maintenance of the O-GlcNAcome catalog: a smart scientific database
Source: Database (Oxford). 2021 Jul 19;2021:baab039. doi: 10.1093/database/baab039 (PMC8288053; doi:10.1093/database/baab039)
Supplement: baab039_Supp [file baab039_supp.zip › clean_Malard2021_Database_appendix.pdf]

Supplementary Materials for:

Automatization and self-maintenance of the *O*-GlcNAcome catalogue: A Smart Scientific Database

Supplementary Figures

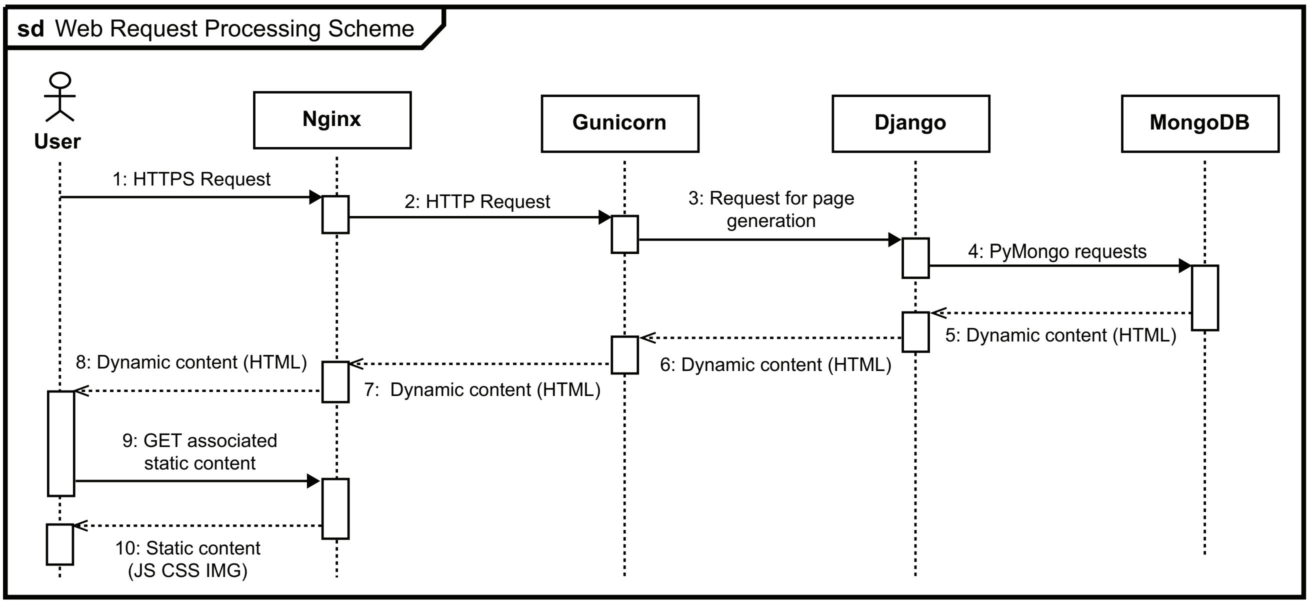

Figure S1: Unified Modeling Language (UML) [11] sequence diagram (sd) of the *O*-GlcNAc Database web request processing scheme. Actor (human symbol), lifeline (horizontal rectangle), activation box (vertical rectangle), synchronous message (solid line) and reply (dashed line) are highlighted per UML conventions. The numbering (1-10) represents the actual sequence of interactions and messages from the actor HTTPS request (1) to the static content response (10).

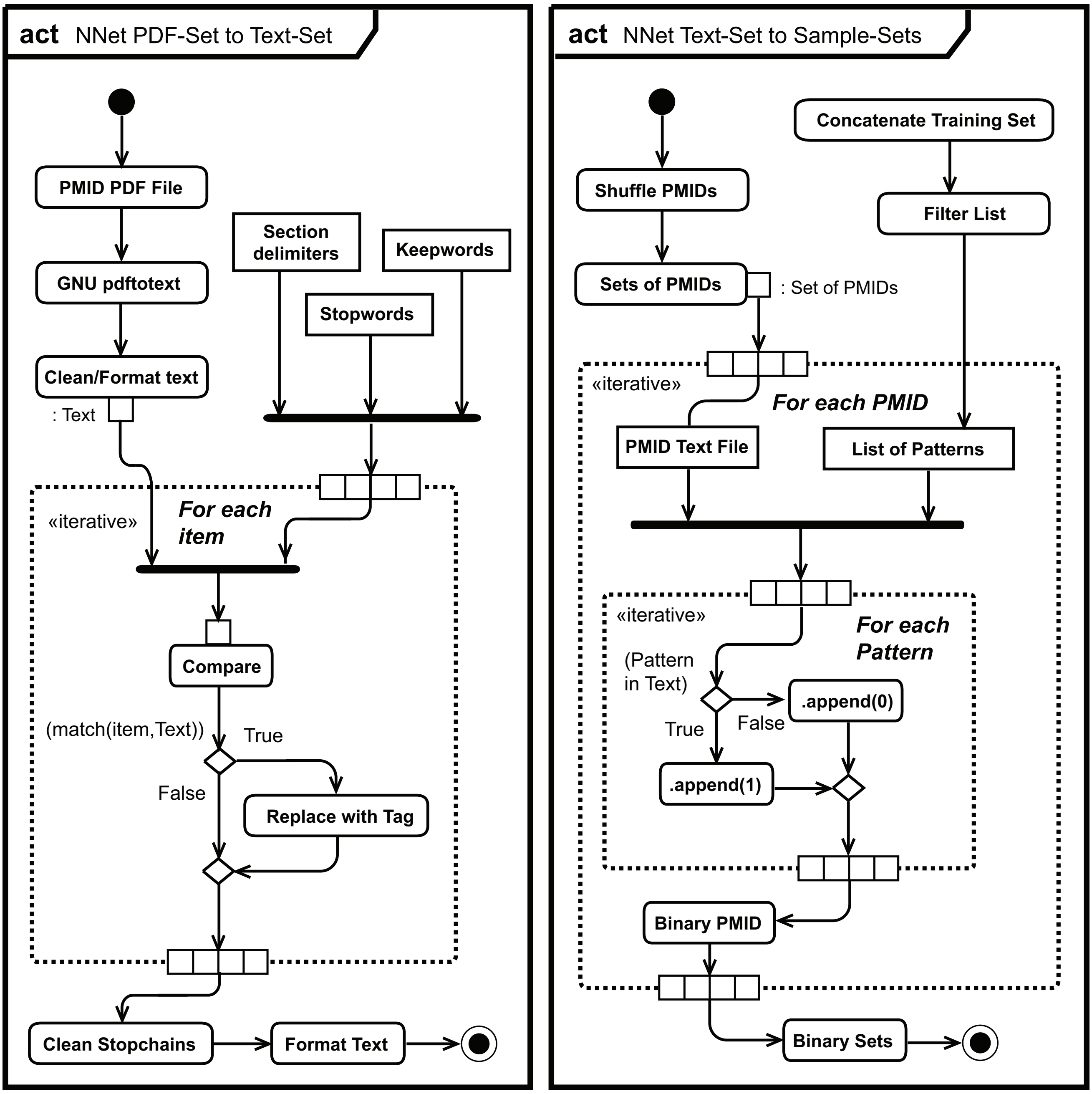

Figure S2: Unified Modeling Language (UML) [11] activity diagram (act) of the *NNet PDF-Set to Text-Set* (left panel) and *NNet Text-Set to Sample-Sets* (right panel) routine. Initial state (black circle), actions (rounded rectangle), list objects (rectangle), join (bold bar), decision and merge (diamond) as well as final state (black circle) are highlighted per UML conventions. Accordingly, UML expansion regions $\ll$ *iterative* $\gg$ represent for loop (left) or nested for loops instructions (right). *Left panel:* Initial state is a list of PMIDs (n = 1 340) which represents a list of PDFs. For each PDF in list, the raw text is extracted using GNU pdftotext, cleaned and appropriately formatted. For each item (word or regular expression) in the list (section-delimiters, stopwords or keepwords), the condition *(match item with text)* is evaluated. If *True*, the current item is replaced by an appropriate tag. Sections of interest (title, abstract, results and discussion) are extracted. Words in the text that were not tagged or contain a *stop* tag are removed to yield period-separated expression patterns made of tags. The final state is a set of text files. *Right panel:* Initial state is a list of PMIDs (n = 1 340) which represents a list of Text. This list is shuffled and sliced to obtain training (n/2), testing (n/4) and validation (n/4) Text-Sets. Samples in the training set are concatenated to yield a dictionnary with expression patterns as keys and their count as values. Patterns with count greater than a cutoff (c = 3) are then included in a list (length = 1 252). For each sample in each set, the corresponding text is read. For each pattern in the list of patterns, the condition *(pattern in text)* is evaluated to 1 if *True* and 0 if *False*, then the Boolean is appended to the input list describing the current sample. This input list, which is 1D array (length = 1 252) containing Booleans, is then appended to the relevant training, testing or validation Sample-Sets. Each Sample-Set in a 2D array of shape (m,1 252) with *m* the number of samples in a given set. The final state is the ensemble of training, testing and validation Sample-Sets.

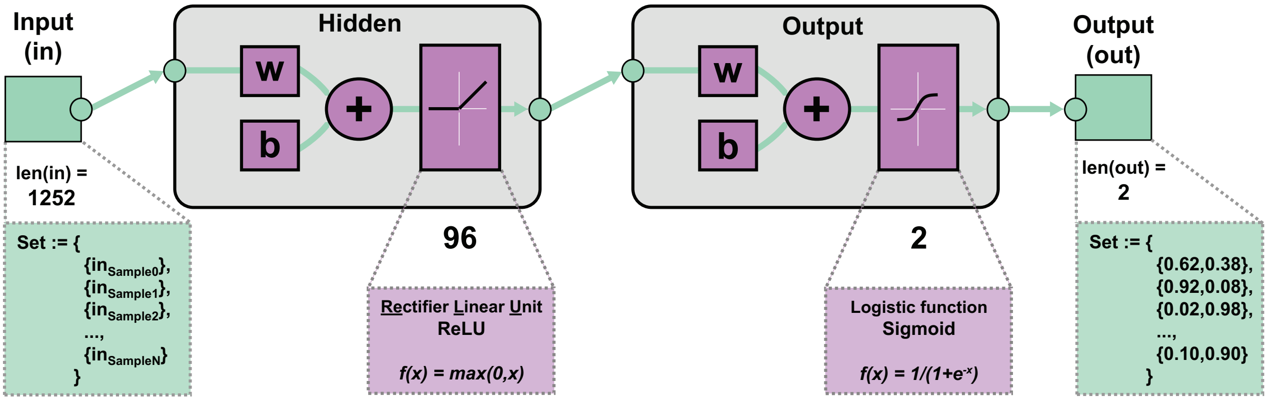

Figure S3: Matlab-style representation of the logistic binary classifier used to predict literature items containing information relative to *O*-GlcNAc protein identification. The underlying NN and layers are highlighted with the input layer containing 1 252 nodes (green square, left), the hidden layer containing 96 Rectifier Linear Units (ReLu) and the output layer with 2 nodes containing sigmoid functions for a final output as a pair of probabilities such as $[P_{positive}, P_{negative}]$. The weight (w) and bias (b) conditions are shown as well as the sum (+) operator applied before passing through functions from the hidden or output layers. In the diagram, we indicate the direction of the control flow (green arrowhead).

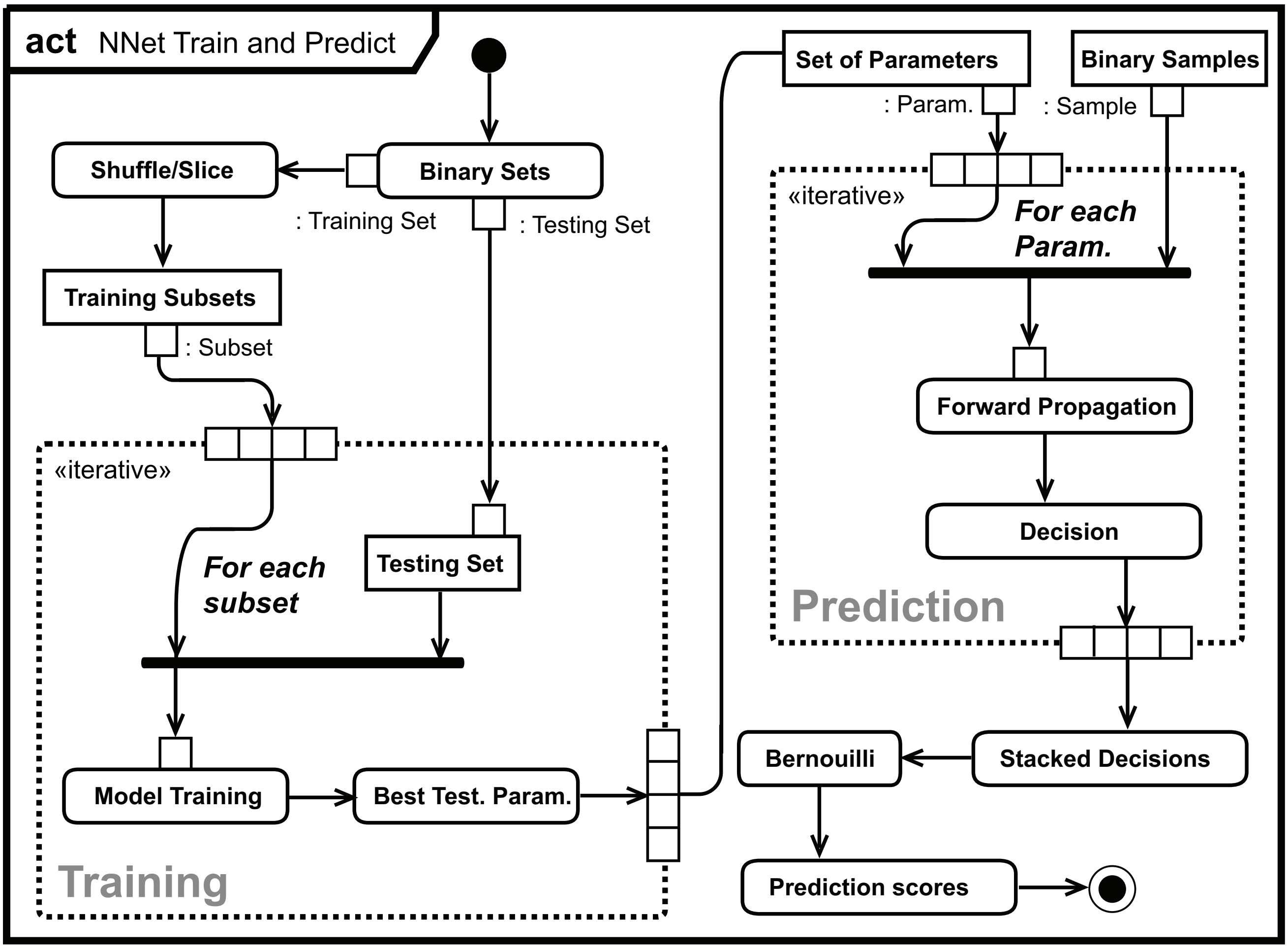

Figure S4: Unified Modeling Language (UML) [11] activity diagram (act) of the *NNet Train and Predict* routine. Initial state (black circle), actions (rounded rectangle), list objects (rectangle), join (bold bar) as well as final state (black circle) are highlighted per UML conventions. Accordingly, UML expansion regions ≪ *iterative* ≫ represent loop instructions. The initial state is a list of sets, each containing samples (Sample-Sets), and of shape (n, in) with n the number of samples in a set and *in* the number of Boolean or inputs (length = 1 252) which describe one sample. The training set (n = 670) is shuffled and sliced, yielding training subsets (n = 355). For each training subset, the regression using a Neural Network designed as a logistic binary classifier proceeds. Weights and bias parameters are saved on disk for the latest best accuracy monitored on the testing set during the regression on each training subset. Series of parameters corresponding to independant models predicted sample labels within all training, testing and validation sets. Corresponding decisions are aggregated and uncertainties computed using a binomial probability formalism. The aggregated model is validated against the validation set and further predicted samples labels on newly published *O*-GlcNAc research articles.

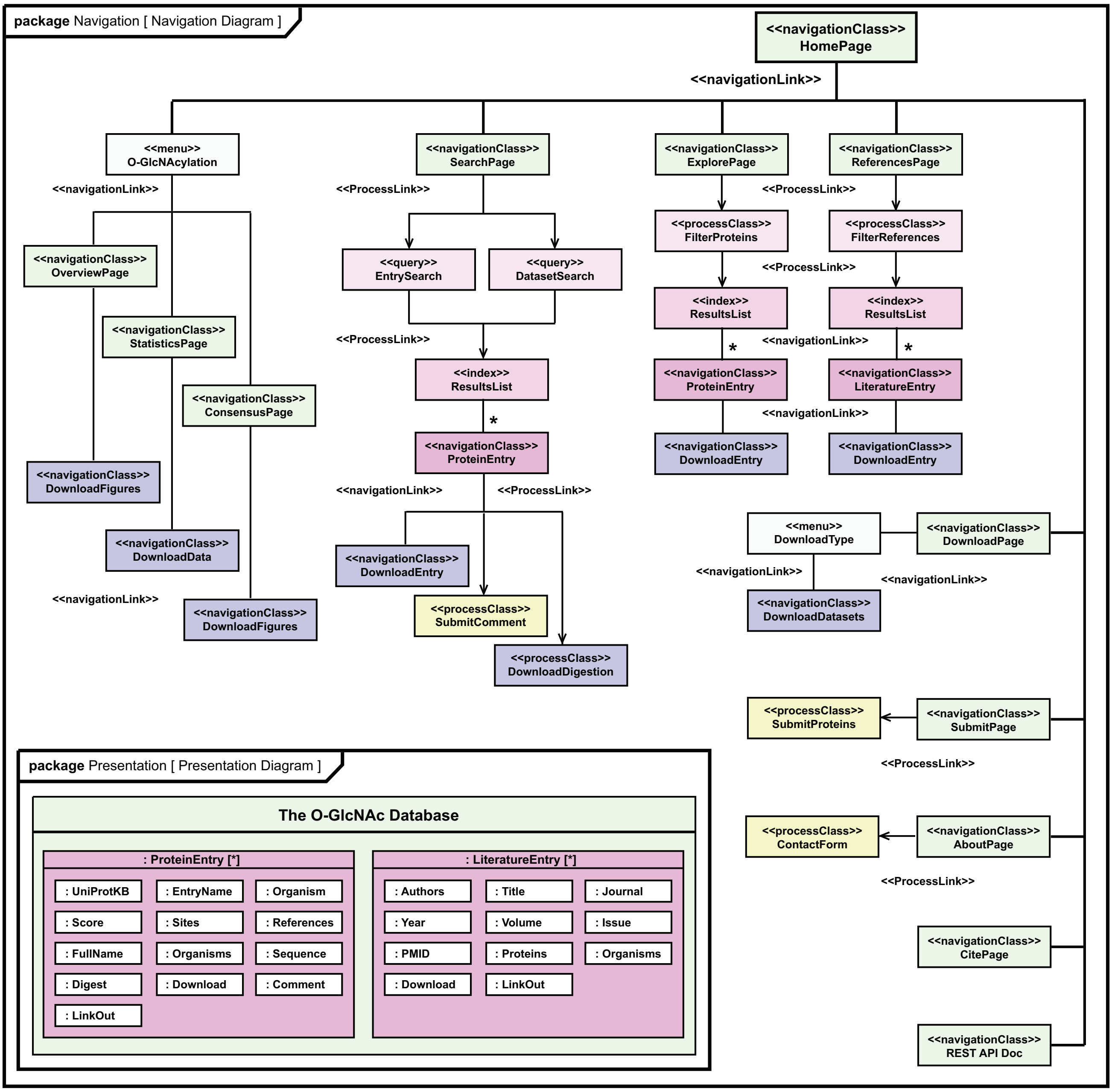

Figure S5: UML-based Web Engineering (UWE) [12] Navigation (main frame) and Presentation (bottom-right frame) diagrams of the *O*-GlcNAc Database (oglcnac.mcw.edu). Static (lightgreen) and dynamic (magenta) navigation classes (*) are highlighted as well as query types and filters (pink), result indexes (lightpink), download links (purple) and submission processes (yellow). In the presentation diagram (bottom-right frame), the navigation classes "ProteinEntry" and "LitteratureEntry" are detailed to show the attributes for each class (white).

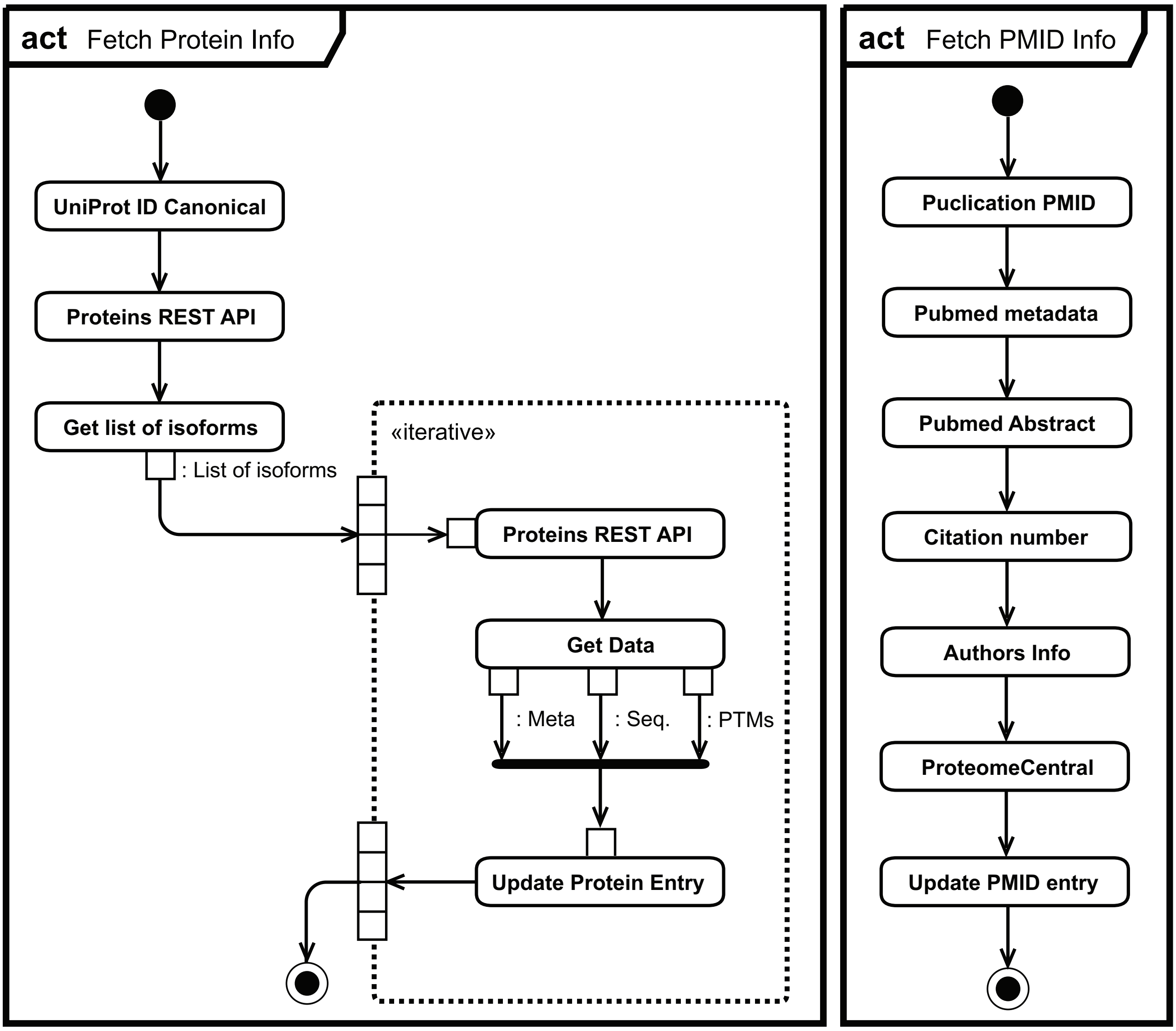

Figure S6: Unified Modeling Language (UML) [11] activity diagram (act) of the *Fetch protein info* (Left panel) and *Fetch PMID info* (Right panel) routines. Initial state (black circle), actions (rounded rectangle), list objects (rectangle), fork and join (bold bars) as well as final state (black circle) are highlighted per UML conventions. Accordingly, UML expansion regions ≪ *iterative* ≫ represent loop instructions. *Left Panel:* The action *Proteins REST API* refers to UniProtKB Protein REST API [50, 51]. *Right Panel:* Pubmed metadata and abstract were retrieved using the Entrez Programming Utilities [52] wheras citation numbers and authors information were obtained from Semantic Scholar API [41, 53]. Command-line tool cURL performed automated search on ProteomeXchange [42] for each PMID.

Supplementary Tables

Library	Source	Use
copy	Module	Shallow and deep copy
csv	Module	Handling csv
datetime	Module	Handling dates
glob	Module	Unix shell style pathname as pattern expansion
gzip	Module	Compression and decompression
inspect	Module	Extract information from live objects
json	Module	Handling json
math	Module	Mathematical functions
operator	Module	Standard operators functions
os	Module	Operating system interfaces
random	Module	Pseudo-random numbers
re	Module	Regular expressions
shutil	Module	High-level file operations
sys	Module	System-specific functions
tempfile	Module	Handling temporary files
time	Module	Handling time
ansi2html	Package	Convert ansi to html
Biopython [54]	Package	Biological computation
json2html	Package	Convert json to html
logomaker	Package	Sequence logo
matplotlib [55]	Package	Plot data
numpy [37]	Package	High-level mathematical functions
pandas	Package	Data analysis and processing
pickle	Package	Read/write data as binaries
Pillow	Package	Handling images
Pygments	Package	Code coloring
PyMongo [21]	Package	Interactions with MongoDB
requests	Package	Handling GET and POST requests
wget	Package	Retrieving files using HTTP, HTTPS, FTP and FTPS
xlrd	Package	Handling xls
XlsxWriter	Package	Handling xlsx

Table S1: Python modules and libraries used in the *O*-GlcNAc Database system. Modules are Python built-in libraries whereas Python package were downloaded from PyPI or GitHub. For security reasons, library versions are not indicated.

Theme	Object	Type	Total	Example	Tagging scheme
Names	List	Litteral	262821	"Lisa"	@_STOPWORDS_@
Journals	List	Litteral	140106	"Bioinformatics"	@_STOPWORDS_@
Chemicals	List	Litteral	106967	"NaCl"	@_STOPWORDS_@
Numerical/Units	List	Litteral	38147	"nmol"	@_STOPWORDS_@
Cities	List	Litteral	23019	"Paris"	@_STOPWORDS_@
Countries/States	List	Litteral	251	"Sweden"	@_STOPWORDS_@
Custom	List	Litteral	69	"Figure"	@_STOPWORDS_@
Biology	List	Litteral	567	"cell cycle"	@_BIOLOGY_@_CELLCYCLE_@@
Glycobiology	List	Litteral	420	"glycolysis"	@_GLYCOBIOL_@_GLYCOLYSIS_@@
Cells	List	Litteral	911	"HEK293"	@_CELLS_@_HEK293_@@
Methods	List	Literal	131	"MALDI"	@_METHODS_@_MALDI_@@
Pronominial	List	Litteral	1	"We"	@_WE_@
O-GlcNAc	List	Regex	4	([^\s]+(NAc)([^\s]+){0,1})	@_OGLCNAC_@_OGLCNACYLATED_@@
Description	List	Regex	5	([^\s]+){0,1}(inhibit)([^\s]+){0,1})	@_DESCRIPT_@_INHIBITED_@@
Conclude	List	Regex	1	(show)([^\s]+){0,1}	@_CONCLUDE_@_SHOWED_@@
Phospho	List	Regex	1	([^\s]+{0,1}phorylat[^\s]+)	@_PHOSPHO_@_PHOSPHORYLATED_@@
S/T-Sites	List	Regex	8	([p]{0,1}[ST][\s]{0,1}[0-9]+)	@_STSITES_@_S34_@@
Peptides	List	Regex	1	([ARNDCQEGHILKMFPSTWYV]{5,})	@_PEPTIDES_@_ALIGRN_@@
Nucleic	List	Regex	1	([ATGC]{5,})	@_NUCLEIC_@_ATGCA_@@
Amino-acids	List	Regex	40	([Aa]la)(nine){0,1}([\s]{0,1}[0-9]+){0,1}	@_AMINO_@_ALANINE_@@
Species	Dictionnary	Litteral	26070 (82396)	{'HUMAN':["Homo sapiens","Human"]}	@_SPECIES_@_HUMAN_@@
Proteins	Dictionnary	Litteral	873434 (5679705)	{'P19332':['TAU', 'Neurofibrillary tangle', 'Microtubuleassociated protein tau']}	@_PROTEINS_@_P19332_@@

Table S2: List of literals, regular expressions (regex) and tags used in the *NNet PDF-Set to Text-Set* routine. The *Object* header defines Python objects. Examples of items in objects are given under the *Example* header with the appropriate Python syntax. We used the python *re.sub()* method with case-sensitive flag all stopwords-related themes as well as proteins and species (if expression does not contain space), *O*-GlcNAc, S/T-Sites, peptides, nucleic and amino-acids. For all other themes and situations, we used case-insensitive match. *Tagging schemes* are shown for each *Theme*, which includes parts conserved (black) and removed (gray) at the end of the routine.

Set	ID	Samples	Positive	Negative
Dataset	NA	1340	670	670
Training set	NA	670	336	334
Training subset	1	335	168	167
Training subset	2	335	167	168
Training subset	3	335	167	168
Training subset	4	335	168	167
Training subset	5	335	168	167
Testing set	NA	335	167	168
Validation set	NA	335	167	168

Table S3: Sets of data and associated class distribution. The number of *positive* and *negative* samples in the whole dataset and in each training, testing and validation sets are shown. Training subsets were iteratively generated by shuffling and slicing half of the training set.

Function	Return	Output (filepath)	Description
fetch_one_UniProtKB(id,filepath=None,pprint=True)	Dictionary	JSON	Fetch UniProtKB Proteins REST API
fetch_one_oglcnacDB(id,filepath=None,pprint=True)	Dictionary	JSON	Fetch The O-GlcNAc Database Proteins REST API
fetch_one_GlyGen(id,filepath=None,pprint=True)	Dictionary	JSON	Fetch RESTful Glygen webservice-based API
fetch_one_PubMed(id,db="pubmed",filepath=None,pprint=True)	Dictionary	JSON	Fetch MedLine/PubMed API using Entrez.efetch
fetch_one_SemanticScholar(id,filepath=None,pprint=True)	Dictionary	JSON	Fetch Semantic Scholar API
fetch_one_proteomeXchange(id,filepath=None,pprint=True)	Dictionary	JSON	Fetch proteomeXchange using GET search request
compute_one_fullDigest(id,protease,filepath=None)	List	JSON	Full digestion of a UniProtKB ID protein sequence
compute_one_partialDigest(id,protease,filepath=None)	List	JSON	Partial digestion of a UniProtKB ID protein sequence
compute_match_aaSeq(id,res,filepath=None)	String	Text	atch residuePosition with UniProtKB ID protein sequence
compute_aln_log2odds(alnpath,organism='HUMAN',filepath=None)	Dictionary	JSON	Compute log2odds for alignment file
draw_one_seqLogo(infile,filepath=None,showplot=True,center_values=False)	None	PNG	Draw sequence logo from compute_aln_log2odds output file
pdf_one_pdf2text(pdfpath,filepath=None,clean=False)	String	Text	PDF to Text conversion with text repair + cleaning.
show_proteases()	List	None	Show list of proteases for digest utils
get_one_sequence(id,filepath=None)	String	Text	Return protein sequence from UniProtKB ID
compute_one_MW(string,filepath=None)	Tuple	JSON	Compute MW of a peptide
get_one_freqAAdict(organism='HUMAN',filepath=None)	Dictionary	JSON	Compute amino-acids frequency table for a given organism
utilsovs_clearCache()	None	None	Clear all data in utilsovs cache

Table S4: Overview of functions in the Python package utilsovs (v0.9.1b). For protein- and literature-related functions, *id* always refers to UniProtKB identifier and PMID, respectively. The full package with documentation is available at github.com/synthaze/utilsovs/.

Organism	Latin name	Proteins	Sites
HUMAN	*Homo sapiens*	7146	9336
MOUSE	*Mus musculus*	3138	1392
TRYCR	*Trypanosoma cruzi Dm28c*	930	0
TRYCR	*Trypanosoma cruzi*	924	0
ARATH	*Arabidopsis thaliana*	500	16
YEAST	*Saccharomyces cerevisiae*	464	0
RAT	*Rattus norvegicus*	447	409
TOXGV	*Toxoplasma gondii*	306	0
TRYCR	*Trypanosoma cruzi marinkellei*	184	0
DROME	*Drosophila melanogaster*	110	27
TRIUA	*Triticum urartu*	110	0
CAEEL	*Caenorhabditis elegans*	68	66
BOVIN	*Bos taurus*	65	7
WHEAT	*Triticum aestivum*	60	2
XENLA	*Xenopus laevis*	27	0
EHRRU	*Ehrlichia ruminantium*	16	0
PLAFB	*Plasmodium falciparum*	15	0
LACPL	*Lactobacillus plantarum*	11	0
PIG	*Sus scrofa*	9	2
HHV8	*Human herpesvirus 8*	6	9
CHICK	*Gallus gallus*	4	3
CHLSB	*Chlorocebus sabaeus*	2	0
CAPHI	*Capra hircus*	2	0
SHEEP	*Ovis aries*	2	0
CLOD6	*Clostridioides difficile*	2	0
CAMDR	*Camelus dromedarius*	2	0
MACMU	*Macaca mulatta*	2	0
RABIT	*Oryctolagus cuniculus*	2	0
PPVRA	*Plum pox potyvirus*	2	4
TOBAC	*Nicotiana tabacum*	2	0
PORGI	*Porphyromonas gingivalis*	2	0
MACFA	*Macaca fascicularis*	1	0
TRIDC	*Triticum dicoccoides*	1	0
RHEAM	*Rhea americana*	1	0
ADE02	*Human adenovirus C serotype 2*	1	0
HCMVA	*Human cytomegalovirus*	1	2
COTJA	*Coturnix japonica*	1	0
CHLAE	*Chlorocebus aethiops*	1	1
LISMO	*Listeria monocytogenes serovar 1/2a*	1	0
TRITU	*Triticum turgidum*	1	0
TRIMO	*Triticum monococcum*	1	0
CANLF	*Canis lupus familiaris*	1	1

Table S5: Number of O-GlcNAcylated proteins and sites. For each organism (UniProtKB ID, Latin name), numerical values for the corresponding number of protein and sites entries are shown.

Supplementary Listings

Results indicate that SP1 is O-GlcNAcylated on serine 120 (1)

NUP62 is O-GlcNAcylated at residue T13 (2)

@_PROTEIN_@ @_OGLCNAC_@_OGLCNACYLATED_@@ @_STSITES_@ (3)

Listing S1: Example of translation from natural language to expression patterns. (1) and (2) are natural language sentences with equivalent meaning but poor similarity. (3) is their common translation into expression pattern following our method.

```python
import numpy as np

olr = 0.25   #Origin learning rate
ilr = olr    #Initial learning rate
k = 0.05     #Decay rate
c = 100      #Cycling parameter
d = 0.95     #Descent parameter

def calc_lr(epoch,ilr):
    #Effective epoch (e) relative to cycling (c)
    e = epoch % c
    #Effective learning rate
    lr = ilr * np.exp(-k*e)
    #Test if next cycle starts at next epoch
    if (e+1) % c == 0:
        #Reduction of ilr for first epoch of next cycle
        ilr = d * ilr
    return lr, ilr
```

Listing S2: Python variables and functions used for learning rate cycling in the *NNet Training* procedure. For each epoch, the learning rate is computed. It exponentially decreases along e, which is the epoch relative to the current cycle. At the end of each cycle, the initial learning rate decrease relative to the constant d.

$$\vec{x} = ([x_1], [x_2], ..., [x_n]) \tag{1}$$

$$p = \frac{1}{n} * \sum_{i=1}^{n} x_i \tag{2}$$

$$p = p \pm 1.96 * \sqrt{\frac{p(1-p)}{n}} \tag{3}$$

Listing S3: Formalism for bootstrap aggregation of predictions from independant models of logistic binary classifier. (1) Ensemble of decision series for each sample. (2) Average of decisions (n = 5) for one sample. (3) Error estimation for one sample to identify ambiguous aggregated prediction.

PROTEIN,[add|remove],[PMID|Site] # Basic syntax scheme

O43823,add,30397120

O43823,add,S21

O43823,remove,S21

P10636,add,22366723,add,S185-8 # With assignment of alternative sequence

PROTEIN,[add|remove],[PMID|Site],[add|remove],[PMID|Site] # Combination PMID and Site in same instruction

O43823,add,30397120,remove,S21

PROTEIN,[add|remove],[PMID;PMID;PMID|Site;Site;Site] # Modification on multiple PMIDs and/or sites

O43823,add,S21;S207

PROTEIN,[delete] # Removal of one entry

O43823,delete

Listing S4: Example of CSV-like instructions in the update file parsed by our automated weekly update routine. Sites are assigned to the canonical protein sequence by default and are validated by our quality control routine. Specific alternative sequences can be assigned with the corresponding notation.

Supplementary Files

File 1: section-delimiters.tar.gz
Dictionnary of regular expressions to detect sections in publications.

File 2: stopwords.tar.gz
Lists of literals to identify and remove non-specific verbiage from PDF to raw text conversion.

File 3: keepwords.tar.gz
Lists of literals and regular expressions for strings of interest.
